# Sampling of non‐Gaussian Ensemble Average Propagators for the simulation of diffusion magnetic resonance images

**DOI:** 10.1002/mrm.70080

**Published:** 2025-09-06

**Authors:** Justino R. Rodríguez‐Galván, Pablo Villacorta‐Aylagas, Susana Merino‐Caviedes, Federico Simmross‐Wattenberg, Carlos Castillo‐Passi, Pablo Irarrazaval, Antonio Tristán‐Vega, Carlos Alberola‐López

**Affiliations:** ^1^ Laboratorio de Procesado de Imagen (LPI) Universidad de Valladolid Valladolid Spain; ^2^ Pontificia Universidad Católica de Chile Santiago Chile; ^3^ Instituto de Investigación Biosanitaria de Valladolid Valladolid Spain

**Keywords:** diffusion MRI, Ensemble Average Propagator, MRI simulators, random sampling

## Abstract

**Purpose:**

(a) To design a methodology for drawing random samples of any Ensemble Average Propagator (EAP) (b) to modify the KomaMRI simulator to accommodate them as realistic spin movements to simulate diffusion MRI (dMRI) and (c) to compare these simulations with those based on the Diffusion Tensor (DT) model.

**Theory and Methods:**

The rejection method is used for random sampling of EAPs: starting from a probability law that is easily sampled, and whose density function *wraps* the target EAP, samples are accepted when they lie inside the targeted region. This is used to sample the EAP as described by Mean Apparent Propagator MRI (MAP‐MRI) and in Spherical Convolution (SC) based on Spherical Harmonics (SH). With this methodology, MAP‐MRI and SC representations are calculated over *in‐vitro* pig hearts images, and a simulation of a pulsed‐gradient spin echo (PGSE) dMRI sequence inside the myocardial wall is undertaken with the KomaMRI simulator.

**Results:**

MAP‐MRI shows better agreement with the actual acquisition than conventional DT‐based simulations, in terms of Mean Squared Errors and correlation with improvements up to 1.7 % for the former and 2.2 % for the latter.

**Conclusion:**

dMRI sequences can be simulated accurately (yet, efficiently) if phantoms with a proper per‐spin description of the diffusion process are made available. Moreover, our findings suggest that the study of non‐Gaussian diffusion of the heart might be feasible, at least *in vitro*.

## 1 INTRODUCTION

MRI simulators constitute a useful tool for educational and research purposes. For the former, clinical demands and equipment cost imply extensive scanner use, so hands‐on sessions for practitioners can hardly be afforded.^1^, ^2^ For the latter, simulations allow users to isolate specific phenomena by selectively removing undesired effects, such as off‐resonance and non‐ideal hardware behavior. Consequently, several MRI simulators have been proposed in the literature since the early days^3^, ^4^ and different computationally efficient simulators are available for different purposes,^5^, ^6^, ^7^, ^8^, ^9^, ^10^, ^11^, ^12^ with some including diffusion.^6^, ^9^, ^13^

Among the diversity of MRI sequences, diffusion MRI (dMRI) has an unparalleled potential to reveal the anisotropy of soft tissues (e.g., the organization of myelinated axons of the white matter of the brain or the muscular fibers of the myocardial wall). This is achieved, among others,^14^, ^15^ by combining the original Spin Echo (SE) sequence with two strong pulsed gradients with the same polarity, plus an inversion pulse in between. This way, the random movement of water molecules during the lapse between the two gradients produces a Diffusion Weighted Image (DWI) whose attenuation depends on the movement direction relative to the orientation of the gradients.^16^ Although applications based on Diffusion Tensor Imaging (DTI) implicitly model this movement as Gaussian,^17^ DTI is an oversimplified model in many situations.^18^

This issue carries over to dMRI simulation: while Gaussian diffusion can be simulated by numerically solving the Bloch‐Torrey's equation,^19^ more general diffusion is challenging. Alternatively, diffusion can be simulated by tuning the proton density (PD) with a diffusion‐driven attenuation model^9^; however, this is only an approximation. Microstructural simulation,^6^, ^13^ on the other hand, relies heavily on an accurate model for the simulated anatomy.

In this work, we aim at simulating non‐Gaussian behaviors in dMRI. Instead of attempting to generalize and solve the Bloch‐Torrey's equation, we (1) design a solution to draw random samples from general 3D EAPs to simulate spin motion during the diffusion time, and (2) extend the capabilities of the KomaMRI simulator^11^ to accommodate this motion. To our knowledge, only one procedure for a similar sampling has been used for a diffusion‐related task,^20^ but not for modeling end‐to‐end spin displacements intended for DWI simulation. We base the description of spin movements on signal representations rather than on biologically inspired models^18^ to attain an anatomy‐agnostic methodology. Specifically, we have focused on two such representations, namely: Mean Apparent Propagator MRI —MAP‐MRI—^21^ and Spherical Convolution —SC— based on Spherical Harmonics —SH—.^22^ However, our approach extrapolates to any other signal representation.

As a proof of concept, we have chosen to simulate diffusion in the myocardium of an *in‐vitro* heart.^23^ reported that high‐order diffusion models, compared to DTI, were unlikely to provide more accurate descriptions due to long TEs together with the T2 signal loss effect. Simulation provides a ground to test the validity of this statement in ideal conditions with a still heart. If in these conditions differences are not observed, the statement seems well founded. Otherwise, room for further research exists when these conditions are not ideal.

## 2 THEORY

### 2.1 The Ensemble Average Propagator in dMRI

Let δ be the duration of the pulsed gradients and Δ the inter‐pulse lapse of the sequence, so that the effective diffusion time is τ=Δ−δ/3. Within τ, water molecules are driven by Brownian motion, and their net displacement $R\in {\mathbb{R}}^{3}$ can be modeled as a 3‐D random variable (RV) whose Probability Density Function (PDF), P(R;τ,D), is dubbed Ensemble Average Propagator (EAP).^16^ For Gaussian diffusion:

(1) $$P(R;\tau ,D)=\frac{1}{\sqrt{{\left(4\pi \tau \right)}^{3}|D|}}\exp\left(-\frac{R^{T}D^{-1}R}{4\tau }\right),$$

where D is the diffusion tensor (DT), the 3×3 symmetric, positive‐semidefinite covariance matrix of the process. Drawing samples from such distribution is straightforward by generating 3 uncorrelated Gaussians with zero mean and unit standard deviation, $S={\left[u,v,w\right]}^{T}:\{u,v,w\}∼𝒩(0,1)$, and computing: $R=D^{1/2}S$. Non‐Gaussian diffusion does not admit a general expression for the EAP, since P(R;τ,D) can be any non‐negative, unit‐mass distribution that fulfills antipodal symmetry: P(R;τ,D)=P(−R;τ,D). Among the many methods aimed at describing arbitrary EAP representations, we will focus on the following two:

- Using **Cartesian** coordinates, the MAP‐MRI formalism draws the EAP as a basis functions expansion in terms of Hermite's polynomials $H_{n}$ (with physicists' convention):^21^
(2) P(R′;τ,D)=∑(even)N=0Nmax∑∑∑n1+n2+n3=N{n1,n2,n3}an1,n2,n3ℋn1(x′;sx)ℋn2(y′;sy)ℋn3(z′;sz):ℋn(t;s)=12n+1/πn!sexp−t22s2Hnts,
  with rotated coordinates ${\left[x^{\prime },y^{\prime },z^{\prime }\right]}^{T}$ and scaling factors $\left\{s_{x},s_{y},s_{z}\right\}$ related to the spectrum of D as:
(3) $${\text{R}}^{\prime }=U^{T}\text{R};\;\;\left[\begin{array}{c} s_{x}\\ s_{y}\\ s_{z} \end{array}\right]=\left[\begin{array}{c} \sqrt{2\;\tau \;\lambda_{x}}\\ \sqrt{2\;\tau \;\lambda_{y}}\\ \sqrt{2\;\tau \;\lambda_{z}} \end{array}\right]\;\;\;:\;\;\;D=U\Lambda U^{T},$$
  with U the matrix of eigenvectors of D and $\lambda_{x}\geq \lambda_{y}\geq \lambda_{z}$ its eigenvalues, hence $\Lambda =\text{diag}\left(\lambda_{x},\lambda_{y},\lambda_{z}\right)$.
- Using **Spherical** coordinates, the EAP can otherwise be expressed as the SC of a fiber‐Orientation Distribution Function (fODF), $\Phi (r),\;\text{r}\in 𝒮\equiv \{\forall \;\text{v}\;\in \;{\mathbb{R}}^{3}:\|\text{v}\|=1\}$, with a rotation‐invariant Gaussian kernel:
(4) $$P\left(R;\tau ,\lambda_{\|},\lambda_{⊥}\right)=\iint_{S}\Phi (v)\;\kappa \left(R,v;\lambda_{\|},\lambda_{⊥},\tau \right)dv,$$
  where $\kappa \left(\text{R},\text{v};\lambda_{\|},\lambda_{⊥},\tau \right)$ has the functional form in Equation (1) for a DT, D, whose main eigenvector and eigenvalue are, respectively, v and $\lambda_{\|}$, and whose two remaining eigenvalues are both $\lambda_{⊥}$. Equation (4) is a spherical mixture model, so that P(R;τ,D) can be sampled by randomly picking an orientation v according to the fODF, Φ(v), then building D from its eigenspace, and finally using the aforementioned procedure to draw a Gaussian sample. The fODF is most often represented as an expansion in the basis of real‐valued, symmetric SH over the manifold 𝒮, ${\tilde{Y}}_{l}^{m}(\theta ,\varphi )$:^22^
(5) Φ(r)≡Φ˜(θ,φ)=∑(even)l=0L∑m=−llϕlmY˜lm(θ,φ)≡∑(even)l=0L∑m=−llϕlm2l+12π(l−m)!(l+m)!Plm(cos(θ))𝒯(mφ),
  where $\text{r}={\left[\sin(\theta )\cos(\varphi ),\sin(\theta )\sin(\varphi ),cos(\theta )\right]}^{T}$, 0≤θ<π, 0≤φ<2π with the usual convention for spherical coordinates, $P_{l}^{m}$ are the associated Legendre polynomials, and 𝒯 is either the sine (m>0), the cosine (m<0), or $2^{-1/2}$ (m=0).

### 2.2 The rejection method for sampling arbitrary PDFs

**FIGURE 1:**
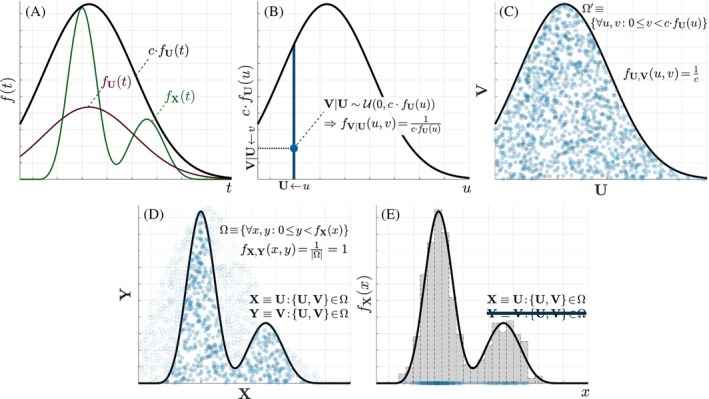
A 1‐D sketch of the rejection method. (A) $f_{\text{X}}$ is the target PDF, $f_{\text{U}}$ is a PDF that can be easily sampled (e.g., the Gaussian in the picture), and c>1 is a constant that ensures $c\cdot f_{\text{U}}(t)\geq f_{\text{X}}(t),\forall t$. (B) For any instance u generated according to $f_{\text{U}}$, a uniformly distributed sample v is generated within the range $\left(0,c\cdot f_{\text{U}}(u)\right)$. (C) By systematically doing so, we get a uniform sampling of the (1+1)‐D variable {U,V} within the domain $\Omega^{\prime }$. (D) The method rejects all samples outside the domain Ω whose upper boundary is defined by $f_{\text{X}}$, which yields a (1+1)‐D variable {X,Y} uniformly distributed in Ω itself. (E) Finally, the component Y is discarded, so that the marginal variable X is distributed according to $f_{\text{X}}(x)$ as we pursued.

Sampling of arbitrary EAPs reduces to the generation of either 3‐D (Cartesian) or 2‐D (spherical) random samples from known PDFs. We will use the rejection method described in,^24^ which is illustrated in Figure 1 for a 1‐D RV, X. The method involves the target PDF of X, $f_{\text{X}}$, an auxiliary PDF $f_{\text{U}}$ of a 1‐D RV U that we know how to sample and a constant c>1 fulfilling $f_{\text{X}}(t)\leq c\cdot f_{\text{U}}(t),\;\forall t$, see Figure 1A. For each instance u of U, the dimensionality of the problem is increased with an auxiliary RV V which is uniformly distributed when conditioned to U, i.e., $f_{\text{V}|\text{U}}(v|u)={\left(c\cdot f_{\text{U}}(u)\right)}^{-1}$, Figure 1B. The 2‐D RV {U,V} is uniform within $\Omega^{\prime }\equiv \{\forall u,v:0\leq v\leq c\cdot f_{\text{U}}(u)\}$, since $f_{\text{U},\text{V}}(u,v)=f_{\text{V}|\text{U}}(v|u)f_{\text{U}}(u)={\left(c\cdot f_{\text{U}}(u)\right)}^{-1}f_{\text{U}}(u)=c^{-1}$, see Figure 1C. Note both U and V|U are trivially sampled (the former by hypothesis, the latter because it is uniform).

The method then rejects all samples {u,v} that lie outside $\Omega \equiv \{\forall x,y:0\leq y\leq f_{\text{X}}(x)\}$, while those inside Ω are accepted as {x=u,y=v}, i.e.,: {X,Y}≡{U,V}|{U,V}∈Ω. As Figure 1D depicts, this results in a 2‐D RV {X,Y} that is uniformly distributed in the new domain Ω, as long as $\Omega \subset \Omega^{\prime }$ (which is guaranteed by the condition on c). It remains to discard the additional dimension Y to retrieve a 1‐D variable X whose marginal PDF is $f_{\text{X}}(x)=\int_{0}^{f_{\text{X}}(x)}1\cdot dy$, see Figure 1E.

This same idea can be extrapolated to any number of RV, by adding 1 additional dimension, provided we can (i) evaluate the N‐D PDF (e.g., using eqs. (2) or (5)), (ii) find a suitable N‐D *wrapper* PDF that we know how to sample (say, Equation 1), and (iii) find a constant c>1 such that the domain of the (N+1)‐D RV expanded from the latter PDF embeds the (N+1)‐D domain of that expanded from the former.

## 3 METHODS

### 3.1 Materials

For the creation of the phantom to simulate with, we had access to an *ex‐vivo* pig's heart left ventricle which presented signs of ischemic cardiomyopathy scar. The acquisitions included dMRI and T1/T2 relaxometry.

Diffusion images were obtained with a Stejskal‐Tanner Pulsed‐Gradient SE (PGSE) sequence (Δ/δ=55.7/16.6ms), followed by an Echo Planar Imaging (EPI) readout. Aside from a baseline acquisition, 192 DWIs were distributed among 6 shells, namely: 300, 600, 900, 1200, 2000, and 3600 $s/{\text{mm}}^{2}$. FOV was set to 140mm, getting 126 lines through SENSE factor 2 resulting in a voxel size of $1.09\times 1.09\times 1.1\;{\text{mm}}^{3}$. Flip angle $=90^{o}$, TE=113.725ms, and TR=50,033ms. The raw images underwent a standard preprocessing pipeline that consisted on denoising and Gibbs ringing correction through Marchenko‐Pastur and PCA techniques,^25^, ^26^, ^27^ followed by correction of eddy currents, motion, and B0/B1 inhomogeneities.^28^, ^29^, ^30^

T1‐relaxometry consisted in a 3D Look‐Locker sequence with 39 different inversion times (TI), starting at 250ms and separated each by 133ms. Flip angle = $5^{o}$, TE=2.88ms and TR=6.043ms. Voxel size was $0.972\times 0.972\times 0.97\;{\text{mm}}^{3}$. T2‐relaxometry used a 3D GraSE acquisition with 18 echoes split by 13ms. TR=1500ms; Flip angle $=90^{o}$; resolution: $0.97\times 0.97\times 0.97\;{\text{mm}}^{3}$. $T_{1}$ and $T_{2}$ maps were obtained from the relaxometry sequences by least‐squares fitting using a custom script. Since a specific PD acquisition did not take place, the spin density map, ρ, was estimated as a function of the diffusion baseline, S, $T_{1}$ and $T_{2}$ assuming an ideal behavior:

(6) $$S\propto \rho \left(1-e^{-TR/T_{1}}\right)e^{-TE/T_{2}}\;\;\Rightarrow \;\;\rho \propto \frac{S}{\left(1-e^{-TR/T_{1}}\right)e^{-TE/T_{2}}}.$$

The protocols indicated above were applied to three slices at different heights in the long axis direction.

### 3.2 Computation of EAP models

From the test data described in Section 3.1, we compute per‐voxel EAPs in both Cartesian and spherical coordinates as described in Section 2.1. To compute the coefficients $a_{n_{1},n_{2},n_{3}}$ in Equation (2) we used the Laplacian‐Weighted MAP‐MRI (MAPL,^31^), with $N_{\max}=6$ as the maximum order for the series expansion, a penalty term for the Laplacian computed via cross‐validation, and 631 positivity constraints defined over the normalized domain of the EAP. Kernel parameters $\lambda_{\|},\;\lambda_{⊥}$ in Equation (4) were computed using the procedure described in^32^ based on the spherical means calculated over each shell. Afterward, the coefficients $\phi_{l}^{m}$ up to degree L=6 in Equation (5) were obtained by SH‐based spherical deconvolution.^22^ In both cases, we used the open‐source Matlab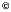 implementations provided in https://www.lpi.tel.uva.es/dmrilab.

### 3.3 Simulation of spins movements

**FIGURE 2:**
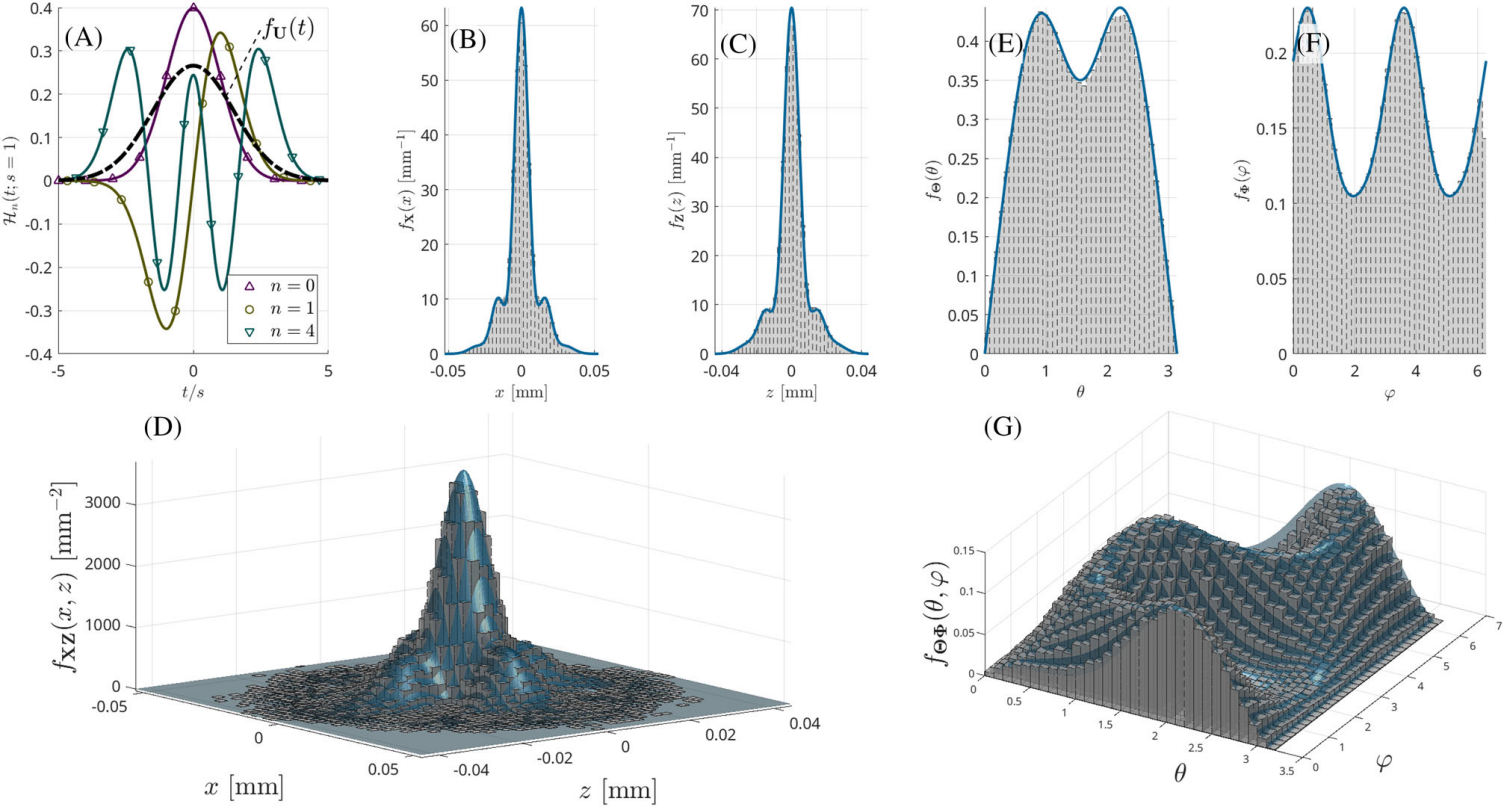
Illustrative examples of random sampling of synthetic PDFs mimicking realistic anatomical configurations ((A) through (D) for MAP‐MRI and (E) through (G) for SC): (A) shows some of the first (normalized) basis functions used to expand the EAP in Equation (2), together with the auxiliary (Gaussian) *wrapper* function $f_{\text{U}}$ used by the rejection method at each normalized coordinate. Histograms of the samples generated according to Equation (2) are compared to the target marginal PDFs in (B) and (C), respectively, for axes ‘x’ and ‘z’ axes, and 2‐D marginalization on the ‘x‐z’ plane in (D). Corresponding results for Equation (5) are the marginal PDFs of each spherical coordinate in (E) and (F) and the joint target PDF in (G).

As in Figure 1A, we need a PDF $f_{\text{U}}$ that can *wrap* the target PDF $f_{\text{X}}$ once multiplied by c. For fODF sampling, since the target PDF is defined in the compact‐supported manifold 𝒮, we use a uniform PDF^1^ in 𝒮 or, equivalently, $f_{\text{U},\text{V}}(\theta ,\varphi )={\left(\sin(\theta )\right)}^{-1}$. For MAP‐MRI sampling, as Figure 2A shows, the basic functions have an unbounded support at each normalized coordinate, since they are calculated as the product of a standard Gaussian PDF, 𝒩(0,1), with a polynomial. Therefore, our *wrapper* function is designed for each normalized coordinate as the product of the 𝒩(0,1) times an exponential $\exp(\kappa \cdot t^{2})$, 0<κ<1/2; this exponential grows faster than a polynomial of any degree, hence the *wrapper* function decays to 0 more slowly than the Hermite functions and it can upper‐bound the target PDF in its entire domain. The product of both exponentials reads (except for constants): $g(t;\nu )=\exp\left(-t^{2}/2\nu^{2}\right)$, with $\nu ={\left(1-2\kappa \right)}^{-1/2}>1$, so that the final wrapper function reads g(x;ν)·g(y;ν)·g(z;ν). In practice, we test discrete values of ν from 1.1 to 1.5 and keep the one leading to the smallest value of c.

Once the *wrapper* function is defined in each case, the constant c is empirically calculated as the maximum of $f_{\text{X}}/f_{\text{U}}$ within a fixed discrete grid (${\left[-5,5\right)}^{3}$ for MAP‐MRI, as Figure 2A suggests, or [0,π)×[0,2π) for fODFs).

For MAP‐MRI, once the random sample ${\left[x^{\prime },y^{\prime },z^{\prime }\right]}^{T}$ is generated, it is rotated back by inverting Equation (3): $\text{R}=U\;{\text{R}}^{\prime }$. For SC, from the random orientation [θ,φ], we build: ${\text{u}}_{1}=[\sin(\theta )\cos(\varphi ),\sin(\theta )\sin(\varphi ),\cos(\theta )]$, ${\text{u}}_{2}=[-\sin(\varphi ),\cos(\varphi ),0]$, ${\text{u}}_{3}={\text{u}}_{1}\times {\text{u}}_{2}$. The DT is calculated as $D=U\Lambda U^{T}$, for $U=[{\text{u}}_{1},{\text{u}}_{2},{\text{u}}_{3}]$, $\Lambda =\text{diag}(\lambda_{\|},\lambda_{⊥},\lambda_{⊥})$. A 3‐D Gaussian sample S with independent components ${\text{S}}_{\left\{x,y,z\right\}}∼𝒩(0,1)$ is created, and finally $\text{R}=D^{1/2}\text{S}$. We are not enforcing strict non‐negativity of the PDFs to sample:^33^ in case negative lobes appear due to numerical issues, the rejection method will crop them by construction, since any candidate value within [0,c·g(x)] will be positive. Figure 2 illustrates the consistency of the method to produce random samples with both approaches.

### 3.4 Simulated diffusion sequence

**FIGURE 3:**
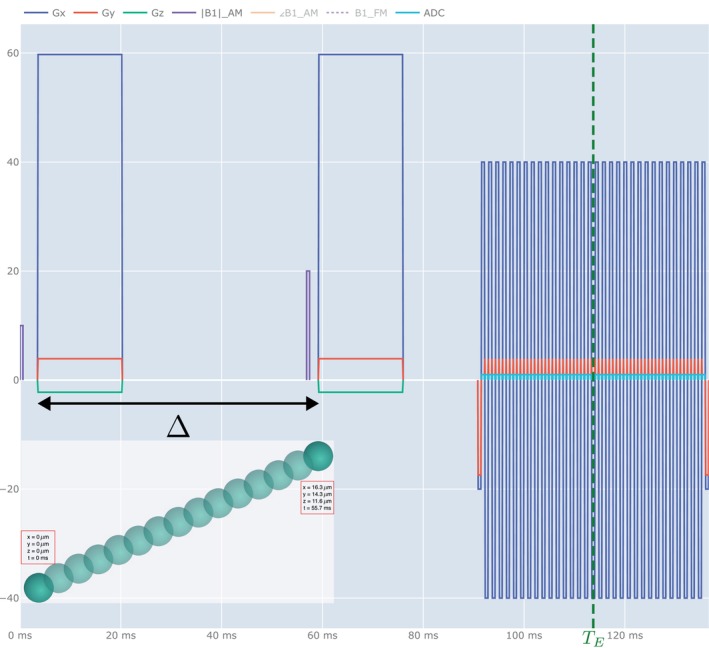
Depiction of acquisition sequence's design delivered by KomaMRI, Δ and $T_{E}$ indicators were included afterward. X‐axis represents time in milliseconds, Y‐axis represents gradient strength in mT/m. RF pulses are represented in purple whilst gradient pulses are represented in blue, red, and green, representing x, y, and z axis encoding, respectively. This particular case represents a PGSE encoded by a gradient with direction [0.9962627247, 0.0780837655, −0.03692572235] at a b‐value of 3600 s/mm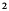. Diffusion motion is set to occur in the midst of both diffusion gradients, taken the effective diffusion time τ as the interval labeled as Δ. The representation in the lower‐left corner illustrates that the spin movement is interpolated between the original and the final position within the Δ interval. The units in both axes do not apply for this representation.

KomaMRI^11^ requires both a phantom and a sequence. The phantom consists of *spins*; for each we have to provide an initial position, a value of the tuple $\left(T_{1},T_{2},PD\right)$ and, if moving, a trajectory. In our case, the information provided by the EAP is the displacement at the end of the Δ‐interval given by an instance of the variable R. All the spins located at the same initial position have the same EAP, although we draw one instance of R per spin. Then, KomaMRI creates a Path ^2^ which conveys the spatial position of each spin for each time point needed in the simulation; with the information provided, the Path is the linear interpolation between the original and the final position of the spin (i.e., the original position plus the displacement). This is illustrated in Figure 3, lower‐left corner. This procedure lets us depart from the *random‐walk* model which, due to the Central Limit Theorem, tends to create Gaussian end‐to‐end displacements for a large number of steps in the walk. Finally, as initial positions we used the 2D pixel grid of each slice. Each grid position within the myocardium was populated with 2000 spins.

As for the sequence, we use PGSE with an EPI readout, see Figure 3. As the current KomaMRI version has no multicoil support, SENSE was bypassed by interlacing two different acquisitions with a blip of difference. Non‐selective excitation is used since the phantom consists of a single slice.

### 3.5 Numerical validation

A three‐fold quality assessment procedure was followed:

1. To check that the simulation was recreating the same orientations, a standard color‐code representation was computed. For that purpose, a DT model was calculated over each model's simulation.
2. To verify that the simulation was consistent with the source data, pixelwise Pearson correlation maps were computed. Specifically, for each shell, we define a vector per pixel consisting of the DWI values across the gradients. For each correlation value, we carry out a Fisher transformation and execute a unilateral test of equality of correlation (i.e., the correlation is at least as high as a tested value). Correlation maps were computed between the simulation and the DWI synthesized by each model, on the one hand, and between the simulation and the acquired DWI, on the other.
3. We computed the normalized mean squared error:
(7) $$NMSE(x,s)=\frac{\overset{N_{g}^{s}}{\underset{j=1}{\sum }}{\left(S_{S}(x,q_{j}^{s})-S_{\text{ref}}(x,q_{j}^{s})\right)}^{2}}{\overset{N_{g}^{s}}{\underset{j=1}{\sum }}S_{\text{ref}}{\left(x,q_{j}^{s}\right)}^{2}}$$
  with x the pixel position, s the shell index, $N_{g}^{s}$ the number of gradients in shell s, $q_{j}^{s}$ the j‐th gradient in that shell, $S_{S}(x,q_{j}^{s})$ the simulated DWI at pixel x for gradient $q_{j}^{s}$ and EAP of the selected model, and $S_{\text{ref}}$ is the DWI value of the reference (for the same pixel and gradient). We used the same two references as for the correlation, i.e., either the DWI synthesized by the selected model or the acquired DWI.

To eliminate the influence of scale factors a normalization of the simulated DWIs with respect to the original acquisition was made beforehand.

## 4 RESULTS

**FIGURE 4:**
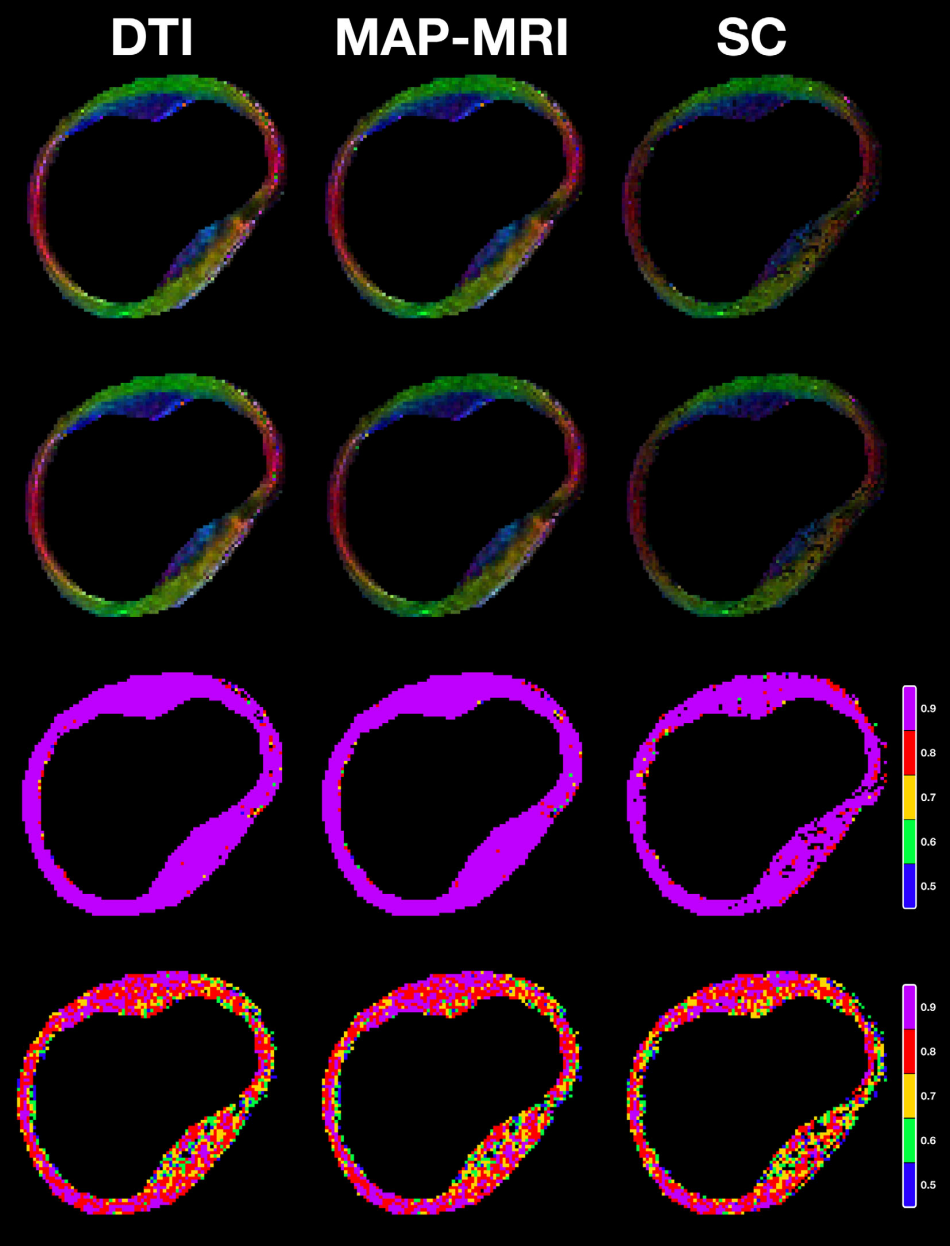
Color orientation maps and color correlation maps. Beginning from the left, first column represents DTI model results, second column MAP‐MRI, and last SC for each row. First row represents color orientation maps for shell of b‐value 600. Second row represents color orientation maps for shell of b‐value 2000. Each map is obtained by adjusting a DTI model to the simulated DWIs in the indicated shell (using the EAP for the simulation corresponding to each model). The last two rows represent correlation maps against the respective model and the original acquisition, respectively, for the 2000 s/mm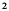 shell. Color code follows color temperature, being blue a correlation coefficient of at least 0.5, green ρ of at least 0.6, yellow ρ of at least 0.7, red ρ of at least 0.8, and purple ρ of at least 0.9.

**FIGURE 5:**
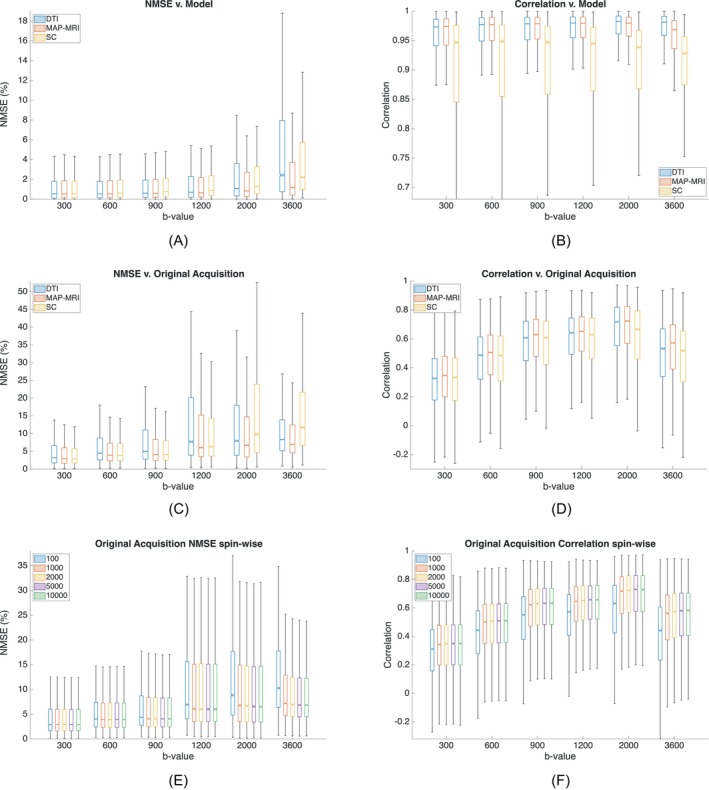
Boxplots comparing point‐to‐point metrics aggregated across three slices. Left column, subfigures (A,C,E): NMSE. Right column, subfigures (B,D,F): correlation. In all the figures, the horizontal axis represents the b‐value. First row, subfigures (A,B) compare each simulation result with its corresponding model. Second row, subfigures (C,D) compare simulation with the original acquisition. Bottom row, subfigures (E,F) illustrate the metrics results for MAP‐MRI and the original acquisition parameterized by the number of simulated spins —from 100 to 10 000— per grid position.

Results are presented in Figures 4 and 5. The former shows color‐orientation maps following the standard RGB code for DT —red for right‐left, blue for inferior‐superior and green for anterior‐posterior— in the two first rows and color‐correlation maps in the last two. As for the orientation maps, the first row corresponds to b‐value 600 and the second one to b‐value $2000\;{\text{s/mm}}^{2}$. Correlation maps use a color temperature code, following blue‐green‐yellow‐red‐purple for intervals [0.5,0.6) until [0.9,1] with a step of 0.1 (see the color bars on the right‐hand side). Each pixel shows a color value that corresponds to the maximum correlation accepted by the hypothesis test. Black pixels are those for which the correlation is not at least 0.5. Each column depicts results for a different diffusion model, as the legend shows.

Figure 5 depicts boxplots of both NMSE (left column) and correlation (right column). In all the cases, the variable in the horizontal axis is the b‐value of each shell. The first row shows boxplots between the simulated DWI and the DWI generated by each diffusion model while the second row compares simulation and actual DWI values. The third row shows the NMSE and the correlation for MAP‐MRI with respect to the actual values parameterized by the number of spins in the simulation.

## 5 DISCUSSION AND CONCLUSIONS

The color‐orientation maps from Figure 4 confirm the coherence of the simulation for the three models (DTI, MAP, and SC). The correlation maps with respect to the DWIs synthesized from each model EAP (third row) show that DTI and MAP‐MRI simulations closely follow their respective models while SC shows scattered areas with lower correlation. Since models are estimated from noisy data —which explains isolated model mismatches— these results mean that the simulation, in essence, does as expected. As for correlation with respect to the acquired DWIs, values are lower although without obvious visual differences between them.

The boxplots in Figure 5 show a similar pattern to that shown in Figure 4. Taking as a reference the model —subfigures (A) and (B)— the three diffusion schemes show similar behaviours, although SC shows a worse fit. In comparison with the original acquisition —subfigures (C) and (D)— MAP‐MRI is consistently better than DTI and SC for both NMSE and correlation for all the shells. Hence, MAP‐MRI was selected for comparing performance parameterized by the number of spins; the results in subfigures (E) and (F) show that the improvement in NMSE and correlation with the number of spins grows for higher shells. In terms of NMSE, 2000 spins seem a sensible trade‐off between performance and computational complexity. We have used this number in the experiments referred to above.

In terms of computation time, EAP sampling and acquisition simulation must be differentiated since they are carried out in CPU and GPU, respectively. The number of grid positions per slice is 1757, 1739, and 711, respectively, for slices 1 through 3. The computation of 2000 samples per grid position took on the order of 7–8 ms in both an AMD Epyc 7513 and an Apple M1‐Pro processor. As for simulation, each grid position took 27 ms in an NVIDIA RTX A5000. Overall, each DWI took roughly 1 min for slices 1 and 2.

Overall, we have chosen two non‐Gaussian diffusion models, which make use of both Cartesian and spherical coordinates, and we have shown that both the EAP sampling and the spin‐oriented simulation with a PGSE‐EPI sequence provide acceptable simulation results for a wide range of b‐values. Moreover, this methodology shows generality so it carries over to other diffusion models and anatomical locations. As for the diffusion models themselves, MAP‐MRI has shown better performance than both DTI and SC. This leaves room for further research on the suitability of higher order diffusion models in the cardiac case. Accordingly, simulation would provide means to anticipate the result in non‐ideal hardware conditions, presence of *off‐resonance* effects, usage of different gradient waveforms or, generally speaking, of different sequences. The limitation is that the simulation remains valid as long as the diffusion time coincides with the one that gave rise to the EAP. This is the price to pay to avoid microstructure in the simulation.

## CONFLICT OF INTEREST STATEMENT

The authors declare there is no conflict of interest.

## Data Availability

The methods for random sampling of the EAP as represented by MAP‐MRI, and the ODF represented with SH expansions, have been coded as multi‐threaded C++ code interfaced to Matlab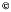 (mex functions), and are publicly available as part of the open‐sourced dMRI‐Lab toolbox: http://www.lpi.tel.uva.es/dmrilab. The particular functions that implement these methods are, respectively, mapl2samples.m and shodf2samples.m.

## ENDNOTES

^1^
This is equivalent to uniformly choosing random 3‐D orientations r, which is trivially accomplished by generating 3‐D Gaussian samples with independent components R∼𝒩(0,1), then computing r=R/‖R‖.

^2^
See https://juliahealth.org/KomaMRI.jl/stable/tutorial/06‐DiffusionMotion/.
